# Evaluation of therapeutic radiographer target volume contouring for magnetic resonance image guided online adaptive bladder radiotherapy

**DOI:** 10.1016/j.ctro.2025.100994

**Published:** 2025-06-17

**Authors:** Bethany Williams, Jonathan Mohajer, Sophie E. Alexander, Helen Barnes, Francis Casey, Joan Chick, Alex Dunlop, Ryan Fullerton, Trina Herbert, Robert Huddart, Sarah A. Mason, Adam Mitchell, Jayde Nartey, Simeon Nill, Priyanka Patel, Shaista Hafeez, Helen A. McNair

**Affiliations:** a The Royal Marsden NHS Foundation Trust, London, UK; b The Institute of Cancer Research/The Royal Marsden NHS Foundation Trust, London, UK; c The Joint Department of Physics, The Institute of Cancer Research and The Royal Marsden NHS Foundation Trust, UK

**Keywords:** Online adaptive bladder radiotherapy, MR Linac, RTT contouring

## Abstract

**Background and purpose:**

One barrier to wider clinical implementation of online MRI-guided radiotherapy (MRIgRT) on the MR Linac (MRL) is resource intensity. Specifically, the requirement for a clinical oncologist/radiation oncologist (CO/RO) to perform online contouring each fraction. We report an evaluation of therapeutic radiographer (RTT) online contouring for patients receiving whole bladder MRIgRT.

**Materials and methods:**

RTTs undertook a contouring training programme. RTT and CO/RO clinical target volume (CTV) contours from 95 fractions were assessed using dice similarity coefficient (DSC), hausdorff distance (HD), mean distance to agreement (MDA), sensitivity and specificity volume metrics on the Raystation treatment planning system (TPS) (RaySearch Laboratories). Additionally, CTV DSC was evaluated with respect to a simultaneous truth and performance level estimation (STAPLE) generated in ADMIRE (Elekta AB, Stockholm, Sweden). In dosimetric analysis (Monaco, Elekta AB), online adaptive treatment plans, which had been generated using RTT-defined contours, were evaluated using contours delineated offline by CO/ROs.

**Results:**

Comparison of RTT versus CO/RO contours found the CTV median (interquartile range) (IQR) for DSC was 0.92 (0.91–0.94), MDA was 0.11 (0.09–0.12) cm, and HD was 0.63 (0.53–0.72) cm, sensitivity and specificity were 0.94 (0.90–0.96) and 0.95 (0.92–0.97) respectively. In dosimetric analysis, 65 % (30/46) plans met optimal PTV coverage of V52.25 Gy > 98 % and all plans met mandatory PTV coverage of V52.25 Gy > 95 %.

**Conclusion:**

Following effective training, evaluation results demonstrate RTT whole bladder CTV contours to be comparable to CO/RO contours. Clinical implementation will release CO/ROs from MRL bladder treatments, reducing resource intensity of online workflows.

## Highlights

• Trained RTT bladder target volume contours were found to have excellent agreement with radiation oncologist contours.
• Intra-professional variability between radiation oncologists and between trained RTTs demonstrated similar variance.
• Adaptive plans optimised on RTT contours result in clinically acceptable target coverage of ‘gold standard’ tumour volume.
• Implementation of RTT contouring could release radiation oncologists from online adaptive bladder radiotherapy workflows.
• Training RTTs now, equips the workforce with skills to verify auto-segmentation in the future.

## Introduction

Conventionally, radiotherapy of bladder cancer has required extensive population-based planning target volume (PTV) margins to account for stochastic deformation in bladder volume and shape, resulting in excessive normal tissue irradiation [[1], [2], [3]]. Developments in cone beam computed tomography (CBCT) based adaptive radiotherapy have aimed to address this, with one solution utilising a ‘plan of the day’ approach, whereby treatment plans with varying PTV margins are selected based on daily anatomy [4,5]. This has facilitated normal tissue sparing and advances in dose escalation, however selected plan conformity can be relatively poor compared to opportunity for deformable registration and auto segmentation in online adaptive radiotherapy (oART) [5].

MRI-guided radiotherapy (MRIgRT) using the Elekta Unity MR Linac (MRL) (Elekta AB, Stockholm, Sweden), combines the improved soft tissue contrast of MRI with daily online re-planning to redefine bladder target volumes for each fraction [[1], [2], [3]], as well as the opportunity to monitor intra-fractional filling with cine imaging [1,2]. Feasibility has since been demonstrated in tumour-focused MRIgRT [2], alongside the potential for reduced target margins, aiming to reduce toxicity and improve patient outcomes [[1], [2], [3], [4], [5]].

In oART workflows, a clinical oncologist/radiation oncologist (CO/RO) is often required to perform online target volume and organ at risk (OAR) contouring, as well as plan approval for each fraction. Emerging evidence supports the upskilling of therapeutic radiographers (RTTs) in target volume contouring [[6], [7], [8]], and other skills required of oART [[9], [10], [11], [12]], facilitating more sustainable MRIgRT, wider clinical implementation, and increased patient access.

To our knowledge there are limited publications reporting evaluation of RTT target volume contouring in bladder MRIgRT; however, RTT online contouring has been successfully implemented for prostate MRIgRT [6]. We report a volumetric and dosimetric comparison of RTT and CO/RO contouring including inter- and intra- professional variability for patients receiving 55.0 Gy in 20 fractions to whole bladder MRIgRT.

## Materials and methods

### Training programme

RTTs undertook a training programme for bladder clinical target volume (CTV) and OAR contouring, developed in collaboration with CO/ROs and medical physicists. The training mirrored a successful programme for prostate MRIgRT contouring [6]. This included educational presentations and videos, a minimum of 10 offline cases contoured (with CO/RO review), five observations of a CO/RO contouring online (minimum three patients), and five sessions of online contouring supervised by a CO/RO (minimum three patients) (Appendix A).

### Bladder online workflow

Patients were instructed to empty their bladder immediately prior to each fraction. Following patient set-up, a 1.1 min T2 weighted (T2w) MRI sequence (MR_SESS_) [13] was acquired and registered to the reference image; for fraction one, this was a planning CT. In the adapt to shape (ATS) workflow previously described [1], the reference structure set was deformably propagated onto the MR_SESS_ using a treatment planning system (TPS) generated deformable image registration. CO/ROs were required to perform online contouring and approve the treatment plan for the first treatment fraction. This plan was then selected as the reference plan for subsequent fractions. In accordance with departmental practice, the fraction one CTV was duplicated, and the copy propagated rigidly (‘CTV_rigid’), to assist in the delineation of the urethra for future treatment fractions. The PTV margin expanded from the CTV was 0.5 cm laterally and inferiorly, 1.5 cm anteriorly and superiorly, 1.0 cm posteriorly.

For subsequent fractions, either a CO/RO or RTT independently contoured the CTV and edited the OAR within a 2 cm expansion of the PTV. After contouring, the selected reference plan was re-optimised using the daily anatomy. Online treatment plan dosimetric criteria (Appendix B) specific to the whole bladder (55.0 Gy/20#) were approved by CO/ROs, allowing a delegated operator (medical physicist) to authorise an online plan. A MR verification (MR_VERIF_) image was acquired simultaneously during plan optimisation and compared to MR_SESS_, to assess target coverage. In the case of intra-fractional bladder filling or anatomical change, correction via previously described alternative adapt to position (ATP) workflow was performed [1].

All RTT-contoured and CO/RO-contoured treatment fractions were timed, from patient entry into the treatment room to beam off, and specifically, from start to end of online contouring. The first fractions were excluded (n = 5) from the timing analysis because they intrinsically required more tasks and were always contoured online by the CO/ROs to be used as the reference plan for the subsequent fractions. These fractions were not excluded from volumetric analysis as regardless of fraction, accuracy of contouring should be attempted consistently based on the MR_SESS_. Equally, despite the use of different reference images in fraction one (planning CT) versus all subsequent fractions (fraction one MR_SESS_), the potential difference in starting point was deemed negligible due to the auto-segmentation of the CTV which was deformably propagated per fraction.

### Contouring guide

In accordance with standard practice, both online and offline contouring were directed by patient-specific contouring guides (Appendix C). This closely follows the guide already clinically implemented for RTT-contoured prostate MRIgRT, created in collaboration with CO/RO [6]. It includes reference image CTV contours and information on tumour location to aid image registration in the ATP workflow. The contouring guide was completed by a RTT and approved by a CO/RO.

### Offline analysis

All MR_SESS_ associated structure sets and online adapted plans were duplicated offline. The CTV structure was reset independently to its original deformably-propagated geometry per fraction. This regenerates the auto-segmentation to the online starting point, to ensure all participants were blinded to the contours of the others. Consistent with online departmental practice, the CTV_rigid structure created fraction 1, was available to all observers offline. In one-to-one offline analysis, the alternate profession to the online contourer (either CO/RO or RTT) was asked to re-contour the same MR_SESS_ replicating the same speed and pressures of the online environment.

### Target volume comparison volume metrics

Interobserver variability (IOV) between RTT online contours and offline CO/RO CTV contours, and vice versa, was assessed using volume metrics on Raystation TPS (RaySearch Laboratories, Stockholm, Sweden, V.12.0.0.932). Metrics included dice similarity coefficient (DSC), hausdorff distance (HD), and mean distance to agreement (MDA). Using the same TPS, sensitivity and specificity were used to report classification accuracy of contoured CTV structures [14]. For the purposes of analysis, the CO/RO contoured CTV per fraction, online or offline, was considered as the “gold standard” due to inherent inter-fractional change in bladder deformation.

CTVs (cm^3^) contoured by RTTs and CO/ROs were compared for each MR_SESS_, per individual patient. Statistical significance was tested using a non-parametric Wilcoxon signed-rank test. In comparison of the CTVs contoured by RTTs and CO/ROs collectively, a linear mixed effect model was used to account for fixed effects (RTT versus CO/RO) and random effects (variability between patients) to test for statistically significant difference in volumes by RTTs and CO/ROs overall.

### Dosimetric analysis

Dosimetric analysis methods to determine the clinical impact of contouring IOV have been previously reported [6]. Using a treatment planning system (Monaco, Elekta AB, Stockholm, Sweden), online adaptive treatment plans, which had been generated using RTT-defined contours, were evaluated using contours delineated offline by CO/ROs. Online dosimetric criteria (Appendix B) were used for reporting.

### Intra- and inter-professional variability

A group of CO/ROs (n = 3) and a group of RTTs (n = 3) were asked to contour the same 15 MR_sess_ images. A simultaneous truth and performance level estimation (STAPLE) [15] was generated in ADMIRE (Elekta AB, Stockholm, Sweden) using the CO/ROs’ contours to create a “gold standard” CTV per MR_SESS_. Variance within and between each professions DSC was measured using the two-way random effects intra-class coefficient (ICC) and Brown-Forsythe’s Test.

## Results

95 of 100 fractions from five patients prescribed 55.0 Gy/20# to the whole bladder were analysed. This included 19 fractions from patient 1 with stage T2/T3b N0 M0, 19 fractions from patient 2, 17 from patient 3, 20 from patient 4 and 5, with T2 N0 M0 urothelial carcinoma. Patient 5 was the only female patient. Five fractions were treated using a conventional linear accelerator due to the MRL not being available, this included one fraction for patient 1 and 2, and three fractions for patient 3. 49 fractions contoured online by one of three CO/ROs were then contoured offline by one of four RTTs. 46 fractions, contoured online by one of four RTTs in CO/RO-independent online treatment, were contoured offline by one of three CO/ROs.

**Fig. 1:**
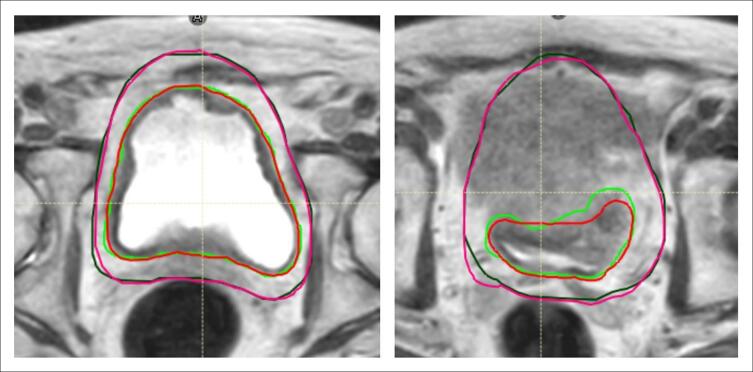
Patient 2 MR_SESS_ transverse plane, at centre of CTV (left), and superior of CTV (right), with online CO/RO CTV (green) and PTV (dark green), and overlaid offline RTT CTV (red) and PTV (pink). For this patient a significant difference was observed in CTV volume (cm^3^) contoured by RTTs versus CO/ROs (*p* = 0.026). (For interpretation of the references to colour in this figure legend, the reader is referred to the web version of this article.)

The median (interquartile range) (IQR) CTV DSC was 0.92 (0.91–0.94), MDA was 0.11 (0.09–0.12) cm, HD 0.63 (0.53–0.72) cm (Table 1). For all patients collectively, no significant difference between CO/RO and RTT CTV volumes (cm^3^) was observed (*p* = 0.673). Individually, four of five patients showed no significant difference in volumes. Patient 2 had a significant difference, with RTT volumes smaller than CO/RO contours (*p* = 0.026) (Fig. 1).

**Table 1:** Similarity metric comparison of 95 CTV structures contoured by RTTs versus CO/ROs. Results are given as median (IQR).

Patient	DSC	MDA (cm)	HD (cm)
1	0.92 (0.89–0.94)	0.09 (0.06–0.11)	0.61 (0.48 – 0.81)
2	0.92 (0.91–0.93)	0.11 (0.10–0.12)	0.66 (0.55–0.71)
3	0.91 (0.90–0.92)	0.11 (0.10–0.13)	0.65 (0.55–0.71)
4	0.96 (0.95–0.96)	0.10 (0.08–0.12)	0.63 (0.53–0.69)
5	0.92 (0.90–0.93)	0.11 (0.09–0.12)	0.63 (0.52–0.72)
Overall	0.92 (0.91–0.94)	0.11 (0.09–0.12)	0.63 (0.53–0.72)

For classification accuracy, the median (IQR) sensitivity and specificity of CTV structures was 0.94 (0.90–0.96) and 0.95 (0.92–0.97) respectively (Table 2).

**Table 2:** Classification accuracy of 95 CTV structures contoured by RTTs compared to CO/ROs. Results given as median (IQR).

Patient	Sensitivity	Specificity
1	0.94 (0.90–0.96)	0.95 (0.88–0.97)
2	0.92 (0.89–0.95)	0.95 (0.92–0.97)
3	0.92 (0.86–0.94)	0.96 (0.93–0.96)
4	0.96 (0.95–0.98)	0.96 (0.95–0.98)
5	0.95 (0.91–0.96)	0.93 (0.88–0.95)
Overall	0.94 (0.90–0.96)	0.95 (0.92–0.97)

In evaluation of intra- and inter- professional variability comparing CTV DSC with respect to STAPLE, the ICC was 0.95 and 0.96 for the group of CO/ROs versus the group of RTTs respectively. Brown-Forsythe’s Test found no significant differences between the variances in the group of CO/ROs versus the group of RTTs (*p =* 0.61).

**Fig. 2:**
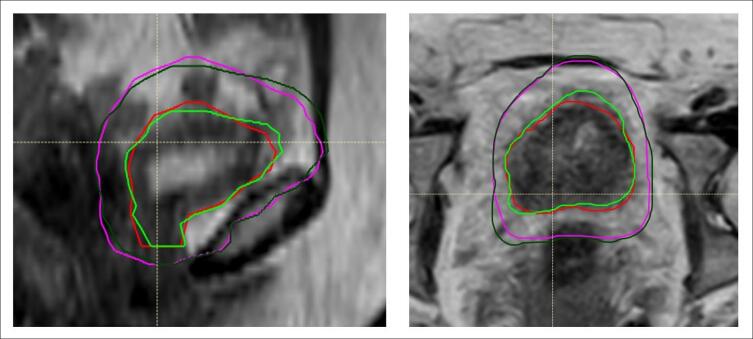

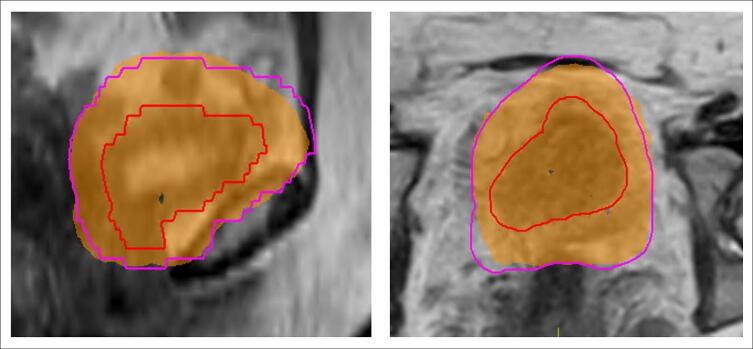
a. Patient 5 MR_SESS_ image in sagittal (left) and transverse (right) planes, overlaid with online RTT CTV contours (red, PTV pink) and offline CO/RO contours (green, PTV dark green). Fig. 2.b. Patient 5, same MR_SESS_ image as presented in Fig. 2.a. with sagittal (left) and transverse (right) planes, overlaid with offline CO/RO CTV (red) and PTV (pink) and 52.25 Gy isodose (orange colourwash) of RTT online adaptive treatment plan. The V52.25 Gy was 96 %, meeting mandatory objective of 95 %. (For interpretation of the references to colour in this figure legend, the reader is referred to the web version of this article.)

**Fig. 3:**
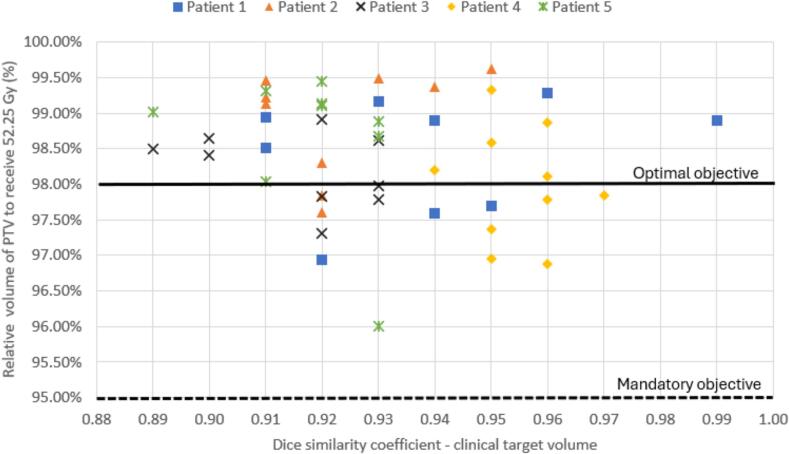
Plot of CTV DSC against PTV dose, in online adaptive plans generated using RTT-defined contours, evaluated using contours delineated offline by CO/ROs.

Of the 46 online RTT-contoured adaptive treatments plans, all plans met optimal CTV coverage of V52.25 Gy > 98 %, when evaluated using clinician-defined offline contours. The relative CTV volume to receive 52.25 Gy was median (range) 99.91 % (99.31–99.99 %). 65 % (30/46) met optimal PTV coverage of V52.25 Gy > 98 % and all plans met mandatory target coverage of V52.25 Gy > 95 %. For those that failed the optimal target coverage objective (n = 16), the PTV V52.25 Gy median (range) was 97.6 % (96.0–98.0 %). An example of this can be seen in Fig. 2b, with CTV IOV observed in Fig. 2a. For patient 2, 77 % (7/9) plans met the optimal PTV objective, and 22 % (2/9) met PTV mandatory objective (Fig. 3), despite that RTT CTV (cm^3^) were demonstrated to be significantly smaller than CO/RO contours.

Recorded total workflow times of 90 fractions were comparable between fractions contoured by CO/ROs and RTTs (Table 3). The time taken to contour online was recorded for 82 of these fractions, as contouring time points were not recorded for 8 fractions. RTTs had a shorter median online contouring time and median total workflow time for three of five patients.

**Table 3:** Median (range) online contouring and total treatment time for CO/RO and RTTs in minutes.

Patient	Contouring time (min)		Total workflow time* (min)	
	CO/RO	RTT	CO/RO	RTT
1	5.6 (3.2–6.3)	5.9 (4.5–9.4)	27.0 (24.5–31.2)	28.3 (25.4–33.4)
2	3.7 (2.1–4.5)	5.1 (3.6–7.3)	24.3 (21.1–22.1)	26.4 (22.1–30.4)
3	6.8 (4.1–7.5)	5.2 (3.8–6.6)	29.4 (26.0–34.7)	27.8 (24.4–32.1)
4	10.2 (7.1–12.8)	8.2 (6.3–12.8)	38.3 (33.7–41.5)	36.3 (33.3–38.9)
5	7.2 (5.1–9.7)	5.8 (4.7–10.5)	29.0 (26.0–42.6)	27.6 (24.9–32.3)
Overall	6.0 (2.1–12.8)	6.0 (3.6–12.8)	28.5 (21.1–42.6)	28.3 (22.1–38.9)

*Time from patient entry into the treatment room to beam off (min).

## Discussion

While previous studies have demonstrated successful implementation of RTT online contouring for prostate MRIgRT [6,8], this is the first study to our knowledge extending this evaluation to bladder MRIgRT. The time taken for RTTs to contour online was similar to CO/ROs, with the median total online workflow time < 30 min, deemed appropriate for PTV margins with intra-fractional filling [2]. Whole bladder CTVs contoured by RTTs were volumetrically comparable to CO/RO contours (median DSC 0.92), consistent with previous prostate CTV evaluations that also showed a median DSC of 0.92 using 117 images from six patients [6]. Alternatively, a prostate CTV DSC of > 0.99 was found with 150 images, eight RTTs and three CO/ROs [8], noting the DSC may have been influenced by subjectivity as observers reviewed RTT contours and chose whether to amend. In contrast, both groups in this study were blinded to the alternate observers’ CTV structures, minimising subjective bias.

Despite the limitations of DSC for use in small volumes and the lack of indication of shape and boundary agreement, DSC remains one of the most frequently used indices in IOV studies [[16], [17], [18]]. Subsidiary volume metrics were used to support the validity of contouring agreement (Table 1). Equally, classification accuracy testing was used to report accuracy of RTT delineation, where sensitivity and specificity of RTT CTV contours were found to be highly accurate (Table 2).

Several IOV studies for anatomical sites other than bladder, conducted structure volume (cm^3^) comparison [6,7] with no significant difference between RTT and CO/RO CTVs [6]. Collectively, this study found no significant difference between each profession’s CTV (*p* = 0.673). Individually however, patient 2, RTT volumes were found to be smaller than CO/RO volumes (*p* = 0.026), possibly due to a more cautious differentiation between the superior aspect of the bladder from the proximal small bowel (Fig. 1). Despite this, this patient’s RTT CTV contours were reviewed and deemed clinically acceptable by a CO/RO and met PTV mandatory objectives in dosimetric analysis (Fig. 3).

It is acknowledged that there is no’ground truth’ when investigating contouring variability [16]. The one-to-one analysis, where the CO/RO CTV is presumed the “gold standard”, is supported by the intra- and inter-professional variability analysis. The CTV DSC ICC for the group of CO/ROs (0.95) and group of RTTs (0.96) demonstrated an excellent degree of agreement in both professional groups (*p =* 0.61), despite inherent bias resulting from the STAPLE volume being generated from CO/RO volumes.

Anatomically, the urethra, bladder base, and superior aspect of the bladder (see Fig. 1) were observed as the areas of greatest variation. Future training efforts should focus on urethral contouring and small bowel contouring on MR sequences, alongside clear patient specific anatomical contouring guidance (Appendix C) [19]. While this study validates its training programme, some extent of inter- and intra-observer variability is an unavoidable outcome of individual judgement. Guidelines, training and clinical experience may help attain and maintain clinically acceptable IOV [19].

Previous studies have attempted to specify a DSC threshold to indicate clinically acceptable dosimetric variation in IOV [6]. Nonetheless, a systematic literature review of 66 IOV studies found data to support a specific DSC was very limited, proposing a minimum requirement for future IOV studies of DSC > 0.7 [16]. In this study no correlation was found between PTV coverage and DSC (Fig. 3). This can be attributed to generous PTV margins for whole bladder compared to prostate MRIgRT and therefore more forgiving dose distributions. A DSC threshold may however be appropriate for smaller PTV margins.

Despite reported IOV differences, 100 % of evaluated plans achieved mandatory 95 % PTV coverage. The evaluated plan with the lowest relative PTV volume (96 %) to receive 52.25 Gy (Fig. 2b) achieved 99.74 % CTV coverage. This could be explained by generous PTV margins, further analysis could be considered for reduced PTV margins or steeper dose gradients in SBRT. Although target coverage during the treatment delivery is a priority, gains of online re-optimisation could be mitigated by use of extensive PTV margins. While intra-fractional filling remains a barrier to PTV margin reduction, this may be addressed with future developments in real-time oART [3]. In the interim, this study demonstrates RTT online contouring to be as efficient as CO/RO contouring in interest of intra-fractional filling.

This study acknowledges offline observers were not subject to equivalent time pressures as online observers. This was balanced by each profession contouring half of analysed contours online and half offline and vice-versa. Equally supported by the inter- and intra- professional analysis where all observers contoured in the offline environment. Both analyses demonstrated excellent agreement. Other limitations of this study include the number of patients and observers, and that it relates to practices in a single specialised department. Further analysis should also include equal variation in patients who are male (n = 4] or female (n = 1), for which the length of urethra inclusion varies. OAR structures were excluded since RTTs were already trained and clinically competent in relevant OAR online contouring [6].

oART techniques are typically resource intensive, unfavourable for health economics and may not be sustainable for wide-scale implementation with current workforce skills [8,20,21]. RTT-contoured online workflows improve efficiency by reducing the expense of CO/RO involvement and enhancing patient throughput due to increased availability of RTTs versus CO/ROs [22]. Given the high validation of RTT contouring in this study, the practice of requiring a CO/RO to contour the first treatment fraction could be phased out, further increasing RTT-contoured oART workflow efficiency.

Internationally, MR Linac centres have successfully implemented CO/RO ‘lite’ workflows, where RTTs lead ATP online workflows for prostate MRIgRT [10]. In support, this study demonstrates that with effective training, RTTs can undertake the role of a CO/RO in online target volume contouring, without compromising a fully adaptive workflow for bladder MRIgRT.

Artificial intelligence (AI) is poised to revolutionise oART. Auto-segmentation will be especially advantageous in the adaptive setting regarding resources and efficiency of oART workflows. Numerous algorithms are demonstrating remarkable contouring agreement [17], but segmentation is not yet readily integrated into current MRIgRT software systems and cannot currently account for clinical decision making. Crucially, this rapidly advancing development necessitates a greater emphasis on training RTTs to ensure that future workforces are equipped to assess the accuracy of auto-segmented structures, thereby enabling safe implementation and guaranteeing high-quality treatment.

The RTT profession is at risk globally; enhancing the scope of practice for RTTs may facilitate staff recruitment and retention [21]. Studies indicate a strong demand for training, including one in which 65 % of 261 UK-based RTTs expressed a desire for training in MRIgRT, with MRI contouring as one of the highest priority needs [8]. This study's validation of RTT capability seeks to support emerging literature [[6], [7], [8], [9], [10], [11], [12], [13], [14], [15]] that emphasises the importance of expanding RTT roles in oART to the multi-disciplinary team, healthcare providers, educational institutions, and policymakers.

## Conclusion

RTT-contoured whole bladder workflows can be effectively implemented on the MR-Linac following appropriate departmental training and assessment. This evaluation demonstrates RTT CTV contours to be comparable to CO/RO contours. Clinical implementation will release CO/ROs from MRL bladder treatments. Multi-centre, international evaluation would enhance the robustness of findings and help facilitate role reallocation on a global scale.

## Patient consent statement

Patients referred for MRI guided adaptive radiotherapy to the whole bladder, 55.0 Gy in 20 fractions, on the MRL were included in this service evaluation, approved by local committee for clinical research. All patients were consented to radiotherapy treatment under the PERMIT CCR4841 trial, approved by research ethics committee (18/WS/0174).

## CRediT authorship contribution statement

**Bethany Williams:** Conceptualization, Methodology, Investigation, Data curation, Writing – original draft, Writing – review & editing, Visualization. **Jonathan Mohajer:** Methodology, Software, Writing – review & editing. **Sophie E. Alexander:** Writing – review & editing. **Helen Barnes:** Writing – review & editing. **Francis Casey:** Writing – review & editing. **Joan Chick:** Writing – review & editing. **Alex Dunlop:** Methodology, Writing – review & editing. **Ryan Fullerton:** Software, Writing – review & editing. **Trina Herbert:** Writing – review & editing. **Robert Huddart:** Conceptualization, Methodology, Writing – review & editing. **Sarah A. Mason:** Formal analysis, Writing – review & editing. **Adam Mitchell:** Methodology, Writing – review & editing. **Jayde Nartey:** Writing – review & editing. **Simeon Nill:** Resources, Writing – review & editing. **Priyanka Patel:** Writing – review & editing. **Shaista Hafeez:** Conceptualization, Methodology, Writing – review & editing, Supervision. **Helen A. McNair:** Conceptualization, Methodology, Writing – review & editing, Supervision, Funding acquisition.

## Declaration of competing interest

The authors declare the following financial interests/personal relationships which may be considered as potential competing interests: This project represents independent research funded by the National Institute for Health Research and Health Education England (HEE/NIHR Senior Clinical Lectureship, Professor Helen McNair, ICA-SCL-2018-04-ST2-002) and supported by the National Institute for Health Research (NIHR) Biomedical Research Centre at The Royal Marsden NHS Foundation Trust and the Institute of Cancer Research, London. The views expressed are those of the author(s) and not necessarily those of the NHS, the National Institute for Health Research or the Department of Health and Social Care. Professor H. A. McNair, B. Williams, J. Nartey and S. A. Mason are funded by The Royal Marsden Cancer Charity and supported by the National Institute for Health Research (NIHR) Biomedical Research Centre at The Royal Marsden NHS Foundation Trust and the Institute of Cancer Research, London. The Royal Marsden Hospital and The Institute of Cancer Research are members of the Elekta MR-Linac Consortium, which aims to coordinate international collaborative research relating to the Elekta Unity (MR- Linac). Elekta (Elekta AB, Stockholm, Sweden) and Philips (Philips, Best, the Netherlands) are commercial members of the MR-Linac Consortium. Elekta financially supports consortium member institutions with research funding, education and travel costs for consortium meetings. Professor R. Huddart reports CRUK Roche and MSD funding for bladder RT trials. S. Alexander reports a relationship with Cancer Research UK that. Research at The Institute of Cancer Research is also supported by Cancer Research UK under Programme C33589/A28284 and C7224/A28724.

## Figures and Tables

**Table:** 

Target	Objective	Level 1	Level 2	Level 3	Level 4
PTV_5500	D99%	>4950Gy	−		<4950Gy
	V5225Gy	>98 %		95–98 %	<95 %
	D50%	>5445Gy	−	<5445Gy	
	D50%	<5555Gy	−	>5555Gy	
	D5%	<5775Gy	−	>5775Gy	−
	D2%	<5885Gy	−	>5885Gy	−

**Table:** 

OAR	Constraint	Level 1	Level 2	Level 3	Level 4
Rectum	V2800Gy	<50 %	≤80 %	>80 %	−
	V4320Gy	<20 %	≤60 %		>60 %
	V5200Gy	<15 %	≤50 %		>50 %
	V5600Gy	<5%	≤30 %		>30 %
Other_Bowel	V2800Gy	<149 cc	≤178 cc	>178 cc	−
	V3980Gy	<116 cc	≤139 cc	>139 cc	−
	V4320cc	<104 cc	≤127 cc	>127 cc	−
	V4800Gy	<91 cc	<115 cc	>115 cc	>130 cc
	V5200Gy	<73 cc	≤98 cc	>98 cc	>120 cc
	V5600Gy	<23 cc	≤40 cc	>40 cc	>50 cc
Femoral_Joint_L	V4320Gy	−	<50 %		>50 %
Femoral_Joint_R	V4320Gy	−	<50 %		>50 %
Normal_Tissue (External − PTV)	D1cc	<5775 Gy	<6050 Gy	>6050 Gy	−

**Figure:**
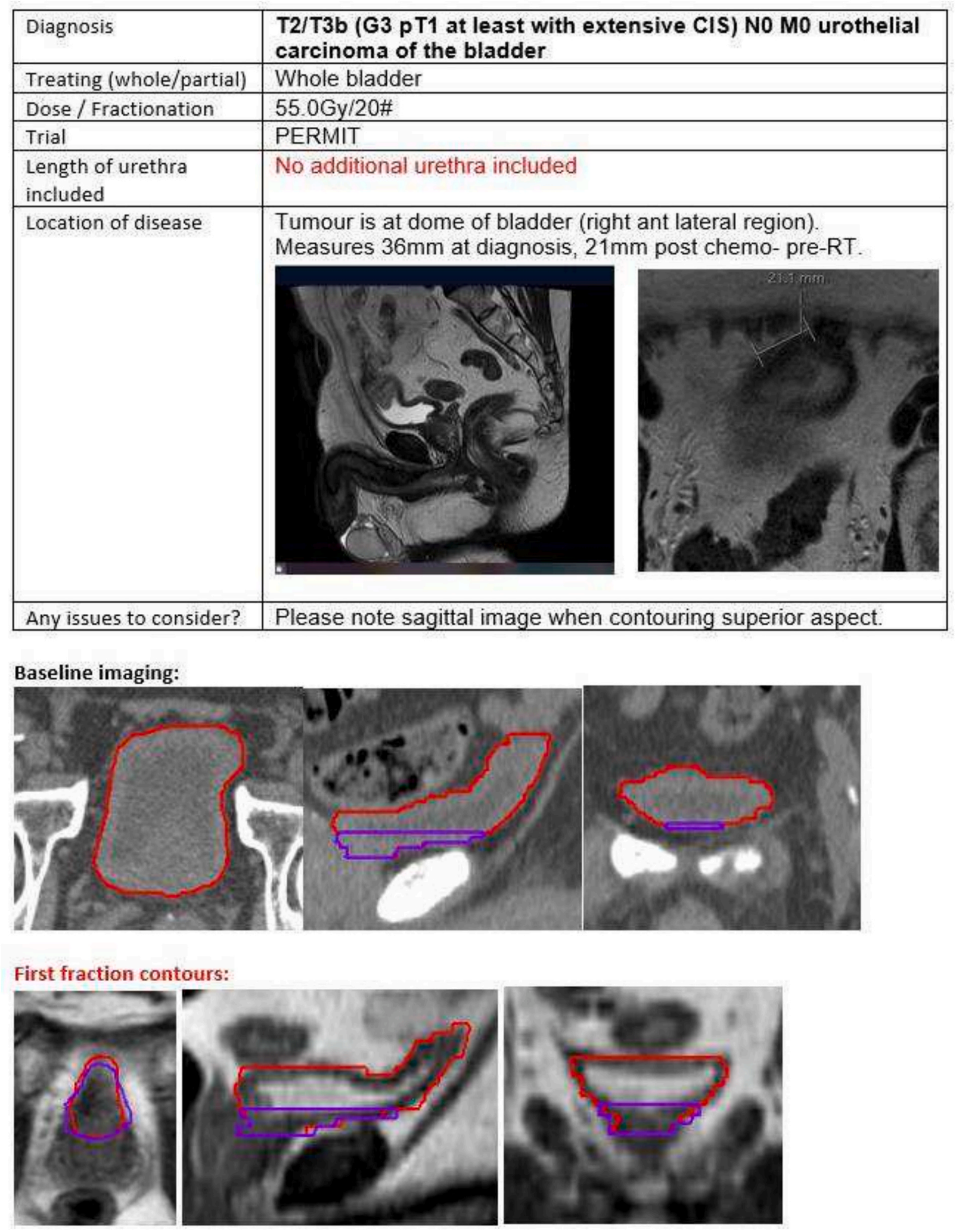


## Appendix A

### Training programme

The training programme, created in collaboration with therapeutic radiographers (RTTs) and clinical oncologist/radiation oncologists (CO/ROs), closely followed an already successfully implemented and validated departmental contouring training programme resources for prostate MRIgRT [6]. RTTs were required to complete the following before independently contouring online:

1. Observation of a virtual presentation and training video:
  a. Bladder cancer aetiology, risk factors and staging.
  b. Bladder anatomy (male and female)
  c. Contouring guidance and considerations specific to contouring bladder target volumes on MRI sequences
  d. Intra-fractional bladder filling considerations
2. Completion of practice cases on a treatment planning system:
  a. Nine offline practice cases contoured independently, all reviewed by a CO/RO in a one-to-one training session
  b. One offline practice case, contoured under review of CO/RO
  c. Five online observations of CO/RO contouring (minimum three patients)
  d. Five online sessions supervised by CO/RO (minimum three patients)

## Appendix B

### Online dosimetric constraints for 20# whole bladder cancer treatments for clinical oncologist/radiation oncologist (CO/RO) independent workflows

This document sets out a framework of target and organ at risk (OAR) online dose constraints for treating 20 fraction whole bladder patients on the MR-Linac (MRL) without a CO/RO present.

These procedures are only valid for CO/RO-independent treatment delivery if there are at least two therapeutic radiographers (RTTs) and one physicist present.

Table 1 displays the Level 1, Level 2, Level 3 and Level 4 online dose constraints for this clinical indication, where:

- Level 1: Optimal constraints met
- Level 2: Mandatory OAR constraints met
- Level 3: Permitted* mandatory constraint violations
- Level 4: Do not treat

*Fractions with one or more Level 3 dose constraint violations may be treated on a one-off basis. After treatment, contact the clinical team to review. After offline review, the CO/RO should provide guidance ahead of the next online fraction which may include updated acceptable online clinical goals for the remaining fractions.

## References

[1] Hunt A., Hanson I., Dunlop A., Barnes H., Bower L., Chick J. (2020). Feasibility of magnetic resonance guided radiotherapy for the treatment of bladder cancer. Clin Transl Radiat Oncol.

[2] Mitchell A., Ingle M., Smith G., Chick J., Diamantopoulos S., Goodwin E. (2022). Feasibility of -focused adaptive radiotherapy for bladder cancer on the MR-linac. Clin Transl Radiat Oncol.

[3] Hijab A., Tocco B., Hanson I., Meijer H., Nyborg C.J., Bertelsen A.S. (2021). MR-guided adaptive radiotherapy for bladder cancer. Front Oncol.

[4] Kong V., Hansen V.N., Hafeez S. (2021). Image-guided adaptive radiotherapy for bladder cancer. Clin Oncol (R Coll Radiol).

[5] Hafeez S., Warren-Oseni K., McNair H.A., Hansen V.N., Jones K., Tan (2016). Prospective study delivering simultaneous integrated high-dose tumor boost (≤70 Gy) with image guided adaptive radiation therapy for radical treatment of localized muscle-invasive bladder cancer. Int J Radiat Oncol Biol Phys.

[6] Adair Smith G., Dunlop A., Alexander S.E., Barnes H., Casey F., Chick J. (2023). Evaluation of therapeutic radiographer contouring for magnetic resonance image guided online adaptive prostate radiotherapy. Radiother Oncol: J Eur Soc Therapeut Radiol Oncol.

[7] Adair Smith G., Dunlop A., Alexander S.E., Barnes H., Casey F., Chick J. (2022). Interobserver variation of clinical oncologists compared to therapeutic radiographers (RTT) prostate contours on T2 weighted MRI. Techn Innov Patient Support Radiat Oncol.

[8] Willigenburg T., de Muinck Keizer D.M., Peters M., Claes A., Lagendijk J.J.W., de Boer H.C.J. (2021). Evaluation of daily online contour adaptation by radiation therapists for prostate cancer treatment on an MRI-guided linear accelerator. Clin Transl Radiat Oncol.

[9] McNair H.A., Hafeez S., Taylor H., Lalondrelle S., McDonald F., Hansen V.N. (2015). Radiographer-led plan selection for bladder cancer radiotherapy: initiating a training programme and maintaining competency. Br J Radiol.

[10] Joyce E., Jackson M., Skok J., Rock B., McNair H.A. (2023). What do we want? Training! When do we want it? Now? A training needs analysis for adaptive radiotherapy for therapeutic RTTs. Radiography.

[11] McNair H.A., Joyce E., O'Gara G., Jackson M., Peet B., Huddart R.A. (2021). Radiographer-led online image guided adaptive radiotherapy: a qualitative investigation of the therapeutic radiographer role. Radiogr (Lond, Engl: 1995).

[12] Hales R.B., Rodgers J., Whiteside L., McDaid L., Berresford J., Budgell G. (2020). Therapeutic radiographers at the helm: moving towards radiographer-led MR-guided radiotherapy. J Med Imag Radiat Sci.

[13] Chick J., Alexander S., Herbert T., Huddart R., Ingle M., Mitchell A. (2023). Evaluation of non-vendor magnetic resonance imaging sequences for use in bladder cancer magnetic resonance image guided radiotherapy. Phys Imag Radiat Oncol.

[14] Udupa J.K., Leblanc V.R., Zhuge Y., Imielinska C., Schmidt H., Currie L.M. (2006). A framework for evaluating image segmentation algorithms. Comput Med Imag Graphics: Off J Comput Med Imag Soc.

[15] Warfield S.K., Zou K.H., Wells W.M. (2004). Simultaneous truth and performance level estimation (STAPLE): an algorithm for the validation of image segmentation. IEEE Trans Med Imaging.

[16] Guzene L., Beddok A., Nioche C., Modzelewski R., Loiseau C., Salleron J. (2023). Assessing interobserver variability in the delineation of structures in radiation oncology: a systematic review. Int J Radiat Oncol Biol Phys.

[17] Matoska T., Patel M., Liu H., Beriwal S. (2024). Review of deep learning based autosegmentation for clinical target volume: current status and future directions. Adv Radiat Oncol.

[18] Mackay K., Bernstein D., Glocker B., Kamnitsas K., Taylor A. (2023). A review of the metrics used to assess auto-contouring systems in radiotherapy. Clin Oncol (R Coll Radiol).

[19] Hunt A., Chan A., Delacroix L., Dysager L., Edwards A., Frew J. (2019). EP-1589 establishing international variation in target delineation using MRI for bladder radiotherapy. Radiother Oncol.

[20] Hehakaya C., van der Voort van Zyp J.R.N., Vanneste B.G.L., Grutters J.P.C., Grobbee D.E., Verkooijen H.M. (2021). Early health economic analysis of 1.5 T MRI-guided radiotherapy for localized prostate cancer: Decision analytic modelling. Radiother Oncol: J Eur Soc Therapeut Radiol Oncol.

[21] Shepherd M., Joyce E., Williams B., Graham S., Li W., Booth J. (2025). Training for tomorrow: Establishing a worldwide curriculum in online adaptive radiation therapy. Techn Innov Patient Support Radiat Oncol.

[22] Xue E, Williams B, Tree A, McNair H, Giorgakoudi K. Cost-consequence analysis of RTT target volume online contouring for prostate MRIgRT. In: ESTRO 2024 – Abstract Book; 2024, p. 2852–3. Glasgow. https://doi.org/10.1016/S0167-8140(24)02694-X.

